# Supplementary material for: Brain Structural and Functional Alterations in Multiple Sclerosis-Related Fatigue: A Systematic Review
Source: Neurol Int. 2022 Jun 8;14(2):506–35. doi: 10.3390/neurolint14020042 (PMC9228847; doi:10.3390/neurolint14020042)
Supplement: Supplementary file 1 [file neurolint-14-00042-s001.zip › neurolint-1735169-supplementary.pdf]

Table S1. Demographics and clinical data of included studies.

Reference	Subjects (women)	Age	Type of MS	MS Duration y	EDSS	T2 LV	Depression Evaluation	Cognitive Evaluation	Clinical Evaluations	Disease Modifying Drugs
Cross Sectional										
[53]	F:5(12) NF:9 (8)	F:43(27-53)* NF:39(23-53)*	RRMS	F:5(1-14)* NF:3(0-9)*	F:3(1-3.5)* NF:2(1.5-3.5)*	NR	Major Depression Inventory score ≤26	DSC F:10 NF:10	**Motor function (CA)** F:98.1(85.1-100)* NF:99.8(96.9-100)*	F: 6 untreated, 10 interferon, 1 glatiramer acetate NF: 4 untreated, 13 interferon
[124]	F:9(21) NF:10(20)	F:41.2(23-54)* NF:40.2(21-62)*	RRMS	F:14.5(1-44)* NF:11.5(1-27)*	F:2.0(1.0-6.0)* NF:1.5(1-6.0)*	F:10.1(13.8) mL NF:11.2(12.1) mL	Chicago Multiscale Depression Inventory below the cut-off level for the Italian population	PASAT F:34.5 NF:34.6	NR	54: interferon beta-1a/1b glatiramer acetate, 6: natalizumab
[60]	F:10(18) NF:11(20)	F:40.5(21-62)* NF:39.6(23-54)*	RRMS	F:13.8(1-44)* NF:11.2(1-27)*	F:2(1-5.5)* NF:1.5(1-6)*	F:8.3(12.9)mL NF:11.2(11.8) mL	CMDI<italian cut off	PASAT-P/PASAT-I: F:22/6 NF: 23/8	NR	NR
[77]	F:15 ModeratelyF: 14 LF:14	F:43(2.9) Moderately F39(3) NF:39(1.7)	RRMS	F:10(1.7) ModeratelyF. 9.2(1.2) NF:8.6(1.9)	F:2(1-4)* ModeratelyF: 2(1-4)* NF:2(1-4)*	F:14(2.5) mL ModeratelyF: 18.8(4.8) mL NF:15.3(5.9) mL	NR	NR	NR	MS: fingolimod.
[50]	F:71 NF:81	F:33.3(8.2) NF:34.4(8.8)	RRMS	F:9.9(7.1) NF:8.7(6.7)	F:3.3(1.8) NF:2.2(1.5)	F:9.2(7.9) cm3 NF:8.8(7.7) cm3	BDI≤18	NR	NR	61: glatiramer acetate 58: natalizumab 33: untreated

[96]	F:2(14)<br>NF:4(9)	F:42.19(11)<br>NF:36.31(9.42)	RRMS	F:11.31(9.3)<br>NF:7.92(4.89)	F:1.37(1.91)<br>NF:1.04(1.35)	NR	NR	NR	**MSS:**<br>F:1.84(2.64)<br>NF:1.31(1.96)<br>**IFS:**<br>F:23.38(0.53)<br>NF:23.88(1.06)<br>**IC-AS**:<br>F:-0.49(0.83)<br>NF:0.64(0.99)<br>**EC-AS**:<br>F:1.09(1.56)<br>NF:1.27(1.63)	NR
[80]	F: 21<br>NF: 17	F:51(44-67)*<br>NF:53(34-62)*	F: 3 RRMS, 11 PPMS, 7 SPMS<br>NF: 3 RRMS, 5 PPMS, 9 SPMS	F:11.90(6.55)<br>NF:11.47(5.30)	F:6.50(5.50-6.50)*<br>NF:6(3-6.50)*	NR	BDI≤19	SDMT:<br>F:39.43(13.73)<br>NF:44.35(12.59)	**HADSanxiety**:<br>F:6.14(3.47)<br>NF:3.88(1.93)<br>**HADSdepression**:<br>F:6.05(2.60)<br>NF:4(2.72)<br>**TAS:**<br>F:52.71(9.25)<br>NF:45.35(11.53)<br>**ESS**:<br>F:3(1-8.50)*<br>NF:1(1-5.50)*	**F**: 8 untreated 4 Dimethyl fumarate, 3 Natalizumab, 2, Interferon-β, 2 Teriflunomide, 1 Methotrexate, 1 Rituximab<br>**NF**: 11 untreated, 3 Natalizumab, 2 Fingolimod, 1 Glatiramer acetate
[99]	F:14 NF:14 (19W 9M)	F:39.1(8.9)<br>NF:37.6(6.6)	RRMS	F:6(1-40)*<br>NF:8(3-22)*	F:1(0-1)*<br>NF:1(0-1)*	NR	NR	NR	NR	NR

[54]	F:3(11)<br>NF:6(8)	F:39.1(8.9)<br>NF:37.6(6.6)	NR	F:6(1-40)*<br>NF:8(3-22)*	F:1(0-1)*<br>NF:1(0-1)*	F:8.9(10.8)mL<br>NF:7.2(4.7)mL	NR	NR	NR	NR
[59]	F:4(11)<br>NF:4(8)	F:37.3(4.0)<br>NF:36.9(7.5)	RRMS	F:3.9(4.1)<br>NF:7.1(3.9)	F:1(0-3)*<br>NF:1(0-2)*	NR	BDI:<br>F:9.3(3.5)<br>NF:6.7(4.2)	NR	**MFIS**:<br>F:42.1(7.3)<br>NF:19.9(8.6)	NR
[39]	F:3(12)<br>NF:4(11)	F:30.4(18-49)*<br>NF:29.9(18-47)*	RRMS	F:2.8(1-7)*<br>NF:3.7(1-9)*	F:1.5(0-1.5)*<br>NF:1.5(0-1.5)*	NR	MADRS≤16	NR	**Pyramidal score**:<br>F:1(0-1)*<br>NF:1(0-1)*	NR
[81]	F:3(19)<br>NF:8(19)	F:31.86(6.84)<br>NF:30.18(6.96)	RRMS	F:7(4.82)<br>NF:5.62(4.79)	F:2.75(1.5-4)*<br>NF:1.5(0-3.0)*	NR	ZDS:<br>F:39.77(8.06)<br>NF33.59(7.47)	PASAT:<br>F:30.90(12.50)<br>NF:35.88(11.93)<br>SDMT:<br>F:47.27(11.80)<br>NF:54.59(13.28)	**EQ5D**:<br>F:0.69(0.20)<br>NF:0.85(0.13)<br>**Pyramidal score**: F:2(1-3)* NF:1(0-2)<br>**9HPT**:<br>F:23.94(4.57)s<br>NF:21.56(3.89)s<br>**T25FW**:<br>F:5.17(0.68)<br>NF:4.48(0.76)	**F**: 7% Intramuscular IFN β-1a 8% Subcutaneous IFN β-1b 4% Subcutaneous IFN β-1a 3% Subcutaneous GA<br>**NF**: 9% % Intramuscular IFN β-1a 8% Subcutaneous IFN β-1b 4% Subcutaneous IFN β-1a 4% Subcutaneous GA 1% Natazumab 1% untreated

[82]	F:1(10)<br>NF:0(6)	F:38(20-49)*<br>NF:26.5(20-47)*	RRMS	F:4.1(0.3-15.5)*<br>NF:3.15(0.7-4.1)*	F:2(0-3)*<br>NF:1.5(1-2)*	F:10.56(1.18-40.51)* cm3<br>NF:3.15(1.40-15.75)* cm3	MADRS:<br>F:7(2-19)<br>NF:9(0-18)	NR	**Mattis score**:<br>F:140(133-142)*<br>NF:139.5(134-142)*	NR
[62]	F:15<br>NF:14<br>(20W 9M)	F:39.2(8.2)<br>NF:37.6(6.6)	RRMS	F:7(1-40)*<br>NF:6.5(2-10)*	F:1(0-1)*<br>NF:1(0-1)*	MS:8.1(1.1-41.7)*ml	MADRS<16	NR	**9HPT**:<br>F:20.2(2.7)<br>NF:20.5(2.5)<br>**Max finger tapping rate**:<br>F:3.4(0.7)<br>NF:3.5(0.5)	NR
[55]	F:32(49)<br>NF:28(38)	F:42.8(11)<br>NF:40.5(10.5)	RRMS:91<br>PPMS:10<br>SPMS:22<br>Others:24	F:13.3(9.1)<br>NF:12.1(6.7)	F:3.5(1-7)*<br>NF:2.5(0-7)*	F:8.9(10)mL<br>NF:8.4(8)mL	MADRS<9	PASAT<br>F:35.9 (0-60)<br>NF:36.5(0-59)	NR	F:57<br>NF:37
[56]	F:26(38)<br>NF:26(33)	F:42.3(10.3)<br>NF:41(10.5)	RRMS:80<br>PPMS:8<br>SPMS:17<br>Others:18	F:13.1(1-44)*<br>NF:11.9(1-32)*	F:2.5(1-7.0)*<br>NF:1.5(0-7.0)*	F:9.7(10.7)<br>NF:8.8(8.3)	MADRS<9	PASAT<br>F:35.7(1-59)<br>NF:37.6(1-59)	NR	**F**: 22 IFN, 8 Copaxone 6 Fg 4 Tys, 3 others<br>**NF**: 15 IFN, 7 Copaxone, 7 Fg, 5 Tys, 0 Others
[61]	F:11(21)<br>NF:10(18)	F:37.72(5.90)<br>NF:34.96(5.87)	RRMS	F:7.44(5.15)<br>NF:5.14(3.69)	F:3.20(1.68)<br>NF:2.21(1.20)	F:6.03(14.02) mL<br>NF:3.16(3.97) mL	CDMI Total score:<br>F:96.14(29.90)<br>NF:64.11(16.23)	NR	NR	NR
[70]	CF:10(27)<br>CNF:2(10)	CF:47.2(10.7)<br>CNF:43.8(10.8)	RRMS	CF:9.1(7)<br>CNF:23(11.2)	CF:3.2(1.5)<br>CNF:4.1(2.7)	NR	BDI:<br>CF:13.4(8.5)<br>CNF:5.7(3.3)	NR	NR	F:82% Immunomodulatory treatment<br>NF:72% Immunomod

										ulatory treatment
[98]	F:4(14)<br>NF:18(24)	F:43.1(6.3)<br>NF:42.9(11.7)	MS:95<br>RRMS:69<br>PPMS:9<br>SPMS:17	F:6.8(6)<br>NF:7.8(8.6)	F:3.4(2.2)<br>NF:3.1(1.8)	NR	BDI:<br>F:9.9(2)<br>NF:5.3(1.8)	PASAT:<br>F:39(12.3)<br>NF:45.4(11.2)	**9HPT**:<br>F:26.5(11)<br>NF:25.4(8.1)<br>**TWT**:<br>F:7.3(3.3)<br>NF:5.8<br>**Alertness without cueing**<br>F:261.1(39.9)<br>NF:246.6(46.2)<br>**Alertness with cueing**:<br>F:248.9(38.6)<br>NF:242.1(43.5)	NR
[72]	CF:20<br>CNF:14<br>(15M 31W)	CF:49.2(7.4)<br>CNF:44(12.9)	RRMS,<br>SPMS	CF:14.4(6.85)<br>CNF:10.4(7.4)	CF:3.8(1.5)<br>CNF:2.5(1.9)	NR	BDI:<br>CF:3.5(3.0)<br>CNF:2.7(2.5)	NR	**MSFC:**<br>CF:-0.2(0.5)<br>CNF:0(0.5)	NR
[71]	CF:19(48)<br>CNF:43(11)	CF:49(10)<br>CNF:43(11)	RRMS,<br>SPMS	CF:12.8(7.3)<br>CNF:10.25(7.1)	CF:3.7(2)<br>CNF:3.3(3)	NR	BDI mental:<br>CF:8(6)<br>CNF:3(3)<br>BDIsomatic:<br>CF:6(3)<br>CNF:4(4)	NR	NR	NR
[63]	F:13(23)<br>NF:37(49)	F:44.3(12.4)<br>NF:35(11.4)	F: RRMS:25<br>Progressive:11<br>NF: RRMS:75<br>Progressive:11	F:13.4(9.8)<br>NF:10.8(6.2)	F:4(0-6.5)*<br>NF:1.5(0-8)*	F:9(8.3)mL<br>NF:5.7(5.3)mL	NR	BRB-N battery<br>Data NR	NR	**F**:7 untreated, 14 Glatiramer acetate/IFN, 15Natalizum

									ab/fingolimod<br>**NF**: 10 untreated, 30 Glatiramer acetate/IFN, 43 Natalizumab /fingolimod
[89]	F: 174<br>NF: 192	F vs NF: 4.62 (2.37, 6.86)**	F: RRMS:93, PMS: 82<br>NF: RRMS:146 PMS: 46	F vs NF: 0.4 (-1.17, 1.97)**	F vs NF: 0.36 (0.12, 0.59)**	F vs NF: _0.03 (0.14, 0.07)**	MADRS: F vs NF: 8.02 (6.35, 9.7)**	PASAT-3: F vs NF: _0.16 (0.47, 0.15)**	NR	**NR**
[52]	F:7(9)<br>NF:4(13)	F:41.1(10.9)<br>NF:38(9.4)	RRMS	F:8.4(3.4)<br>NF:7.9(3.8)	F:1.8(0.6)<br>NF:1.6(0.6)	NR	17-HDRS:<br>F:6.4(4.8)<br>NF:6.1(4.6)	MMSE<24	NR	**MS**: 32 immunomodulatory (interferon-beta-1, glatiramer acetate) or immunosuppressive therapy (azathioprine).
[83]	F:20<br>NF:11<br>(12M 40W)	MS:39(32-43)*	RRMS	MS:9(3-14)*	MS:2.5(2-3.5)*	NR	NR	NR	NR	**MS**: 8glatiramer acetate
[57]	F:9(18)<br>NF:9(12)	F:38.37(7.56)<br>NF:40.67(10.39)	RRMS:44<br>SPMS:5	F:9.87(5.86)<br>NF:8.52(6.46)	F:2.51(1.45)<br>NF:2.24(2.02)	NR	BDI<br>F:12.70(5.96)<br>NF:4.62(3.46)	NR	**tSTAI**:<br>F:55.89(7.58)<br>NF:38.80(9.74)	39 MS: glatiramer acetate, beta-interferon
[99]	F:23(77)<br>ReversibleF:25(75)<br>NF:19(81)	F:48.5(9.2)<br>ReversibleF:51.2(10)<br>NF:51.5(7)	**F**:RRMS:85% SPMS:15%<br>**ReversibleF**: RRMS:90% SPMS:10%	F:18.1(6.9)<br>ReversibleF:15.9(7.6)<br>NF:16.8(8.2)	F:2.3(1.6)<br>ReversibleF:2.1(1.7)<br>NF:1.6(1.6)	NR	CES-D≥16<br>F:14<br>ReversibleF:5<br>NF:1	NR	NR	NR

			**NF**:RRMS:93<br>% SPMS:7%							
[84]	F:20(80)<br>ReversibleF:2<br>6(74)<br>NF:22(78)	F:48.9(8.9)<br>ReversibleF<br>:49.5(9.8)<br>NF:49.4(6.7)	**F**:RRMS:87%<br>SPMS:13%<br>**Reversible**<br>**F**:RRMS:84%<br>SPMS:13%<br>PPMS:3%<br>**NF**:RRMS:89<br>%<br>SPMS:11%	F:17.7(6.6)<br>ReversibleF:1<br>6.9(8)<br>NF:17(8.1)	F:2.3(1.5)<br>ReversibleF:2<br>.0(1.8)<br>NF:1.8(1.0)	NR	CES-D>16<br>F:15.9(8.7)<br>RF:8.8(7)<br>NF:5.8(4.2)	NR	NR	**F**:<br>87%disease-<br>modyfing<br>treatment<br>2monthly<br>intravenous<br>steroids, 1 on<br>immunosup<br>pressant.<br>**ReversibleF**:<br>80%disease-<br>modifying<br>treatment<br>2monthly<br>intravenous<br>steroids<br>**NF**:<br>92%disease-<br>modifyinf<br>treatment, 1<br>immunosup<br>pressant
[58]	F:6(20)<br>ReversibleF:<br>6(19)<br>NF:8(34)	F:48.5(9.2)<br>Reversible<br>F: 51.2 (10)<br>NF: 51.5(7)	F: RRMS:22<br>SPMS: 4<br>Reversible F:<br>RRMS:22<br>SPMS: 3<br>NF: RRMS:39<br>SPMS: 3	F: 17.5(12-22)*<br>Reversible F:<br>13 (12-16)*<br>NF:14(11-21)*	F: 2(1.5-3)*<br>Reversible F:<br>1.5(1-3)*<br>NF:1.5(1-2)*	NR	**CES-D≥16:**<br>F:17<br>Reversible<br>F:8<br>NF:6	NR	NR	NR
[85]	F:8<br>NF:16<br>(7M 17W)	MS:45.4(9.7<br>)	RRMS:20<br>SPMS:4	MS:12.6(8.4)	F:3(1-6.5)*<br>NF:1.5(0-<br>4.5)*	MS:9.2(7.5)c<br>m3	NR	NR	NR	NR
[97]	CF:7(4)<br>CNF:7(4)	CF:46.6(9.3)<br>CNF:40(5.8)	RRMS	CF:9.5(3.8)<br>CNF:6(4.4)	CF:2.5(0-3.5)<br>CNF:1.5(0-3)	CF:9.1(1.4)m<br>L<br>CNF:3.9(6.9)<br>mL	BDI≤10	**PASAT:**<br>CF:81.2(47-<br>118)	NR	NR

								CNF:103.6(73<br>-118)		
[40]	F:4(6)<br>NF:6(8)	F:38(7.7)<br>NF:38.6(8.5)	RRMS	F:8.2(6.2)<br>NF:10.6(6.6)	F:1.5(1.5-2)*<br>NF:1.5(0-<br>1.5)*	F:7.7(5)mL<br>NF:8.9(10.7)<br>mL	MADRS<br>Data NR	NR	NR	NR
[65]	F:12 NF:10<br>(5M 17W)	F:34.1(22-<br>42)*<br>NF:33.1(21-<br>44)*	RRMS	F:6(2-15)*<br>NF:5(2-11)*	F:1(0-3)*<br>NF:1(0-2)*	NR	MADRS<16	NR	**9-HPT**:<br>F:22(19-24)*<br>NF:20(17-<br>22)*<br>**Finger<br>tapping rate**:<br>F:3.2(2.6-4)*<br>NF:3.5(2.5-<br>3.8)*<br>**Foot tapping<br>rate**:<br>F:3.2(2.6-4)*<br>NF:3.1(2.6-<br>3.8)*	NR
[66]	F:1(10)<br>NF:1(12)	F:33.8(6.2)<br>NF:31.2(4.3)	RRMS	F:6(2-12)*<br>NF:6(2-10)*	F:1(0-1.5)*<br>NF:1(0-1.5)*	F:8.1(8.5)ml<br>NF:8.8(6)ml	MADRS<9	NR	**FSS:**<br>F:37.7(7.7)<br>NF:15.6(4.8)	**MS**: 3<br>glatiramer<br>acetate; 21<br>interferon<br>beta
[91]	F:6(14)<br>NF:8(7)	F:38.8(29-<br>65)*<br>NF:38.8(29-<br>60)*	RRMS	F:10.5(2-23)*<br>NF:11.2(0.5-<br>27)*	F:3.5(1.5-5.5)*<br>NF:3(0-5)*	NR	MADRS≤16	NR	NR	NR
[76]	F:14(17)<br>NF:15(17)	F:41(23-63)<br>NF:39.5(27-<br>58)	**F:** RRMS:26<br>SPMS:5<br>**NF**: RRMS:30<br>SPMS:2	F:13(0.6-32)<br>NF: 11.65(0.8-<br>25.5)	F:2.5(1-5.5)<br>NF:2(1-6.5)<br>(median)	F:8.5(8.1)<br>NF:8.7(9.7)	MADRS<br>F:8(1-17)*<br>NF:10(3-17)*	PASAT<br>Data NR	NR	F:9interferon<br>s,<br>5glatiramer<br>acetate,<br>7fingolimod,<br>4Natalizuma<br>b<br>NF:14Interfe<br>rons,<br>2Glatiramer<br>acetate,

										3Fingolimod, 5Natalizumab
[64]	F:17(33) NF:10(19)	F:42.6(11.2) NF:40(9.1)	RRMS	F:12.9(8.2) NF:10.6(7.6)	F:2(1-4)* NF:1.5(0-4)*	F:6.2(7.5)mL NF:5.7(6)mL	MADRS<9	BRB-N<2	**9-HPT**: F:25(6.5) NF:24.7(5.3) **Finger tapping rate**: F:3.5(0.7) NF:3.5(0.7)	NR
[67]	F:8(11) NF:7(9)	F:43(8) NF:42(10)	F: RRMS:9 Others:10 NF: RRMS:10 Other:6	F:10(8) NF:14(8)	F:3.6(1.3) NF:3.9(1.7)	NR	BDI: F:15.6(8) NF:5.2(3.6)	NR	NR	NR
[79]	CF:10(18) CNF:5(9)	CF:50.5(8.8) CNF:42.8(12.8)	**CF**: RRMS:18 SPMS:10 **CNF**: RRMS:12 SPMS:2	NR	T1: **CF**:4.14(1.52) **CNF**:2.63(2.12) T2: **CF**:3.70(1.46) **CNF**:3.42(2)	NR	BDI Data NR	PASAT Data NR	**MSFC**: T1: CF:-0.15(0.53) CNF:-0-01(0.48) T2: CF:-0.02(0.50) CNF:0.09(0.60)	**CF**:15.4%untreated, 53.8%interferons, 30.8%escalated medications **CNF**:46.7%nomedication, 40%interferons, 13.3%escalated medications
[86]	F:43 NF:17 (22M 38W)	MS:36.4(9)	CIS:22 RRMS:28 SPMS:5 PPMS:5	MS:2.66(1-36)*	MS:2(0-7)*	MS:34.57(1.2-175.9)*cm3	Cummings' Neuropsychiatric Inventory, the Hamilton's Depression Rating Scale (<8 points), Hamilton's Anxiety	SDMT, PASAT Data NR	**MSFC**: MS:0.64(0.76) **BRB-N**	NR

							Rating Scale (<6 points			
[68]	F:12<br>NF:12<br>(4M 20W)	F:40.9(8.9)<br>NF:38.7(8.1)	RRMS	MS: 7	F:1.5<br>NF:1.5	NR	BDIS<16	MMSE<24	**MSFC**<br>**MSQOL-54**<br>Data NR<br>Motor performance<br>Data NR	NR
[69]	F:3(7)<br>NF:6(6)	F:27(5.5)<br>NF:25.8(5.4)	RRMS	F:5.5(3.8)<br>NF:5.8(3.8)	F:2.3(0-6)*<br>NF:1.16(0-4)*	F:4.7(2.8)<br>NF:12.4(19.6)	BDI<br>F:3.3(2.3)<br>NF:1(1.8)	NR		NR
[92]	F:6(21)<br>NF:8(9)	F:36.7(9.7)<br>NF:34.5(8.3)	RRMS	F:6.2(4.5)<br>NF:6.4(6.4)	F:2.5(0.8)<br>NF:2(1.1)	NR	BDI-II<br>F:9.7(6.2)<br>NF:3.4(3.3)	PASAT:<br>F:48.8(8.4)<br>NF:52.2(5.3)<br>SDMT:<br>F:53.5(9.3)<br>NF:55.4(12.3)	**PSQI**:<br>F:5.7(4.5)<br>NF:4.4(1.7)<br>**ESS**:<br>F:9(3.5)<br>NF:6.8(4.3)<br>**JTHFT**:<br>F: 37.9(5.1)<br>NF:37.3(2.4)<br>**9-HPT**:<br>F:15.9(2.1)<br>NF:16(2)	NR
[93]	F:34<br>NF:26	F:42.12(6.9)<br>NF:38.09(10.2)	F:RRMS:26<br>SPMS:8<br>NF:RRMS:20<br>SPMS:6	F:10.59(7.3)<br>NF:10.35(9.5)	F:3.81(2.2)<br>NF:2.69(2.2)	F:13.44(13.4)cm3<br>NF:11.24(13.2)cm3	NR	NR	NR	NR
[87]	F:25<br>NF:197	F:39(9.18)<br>NF:34(9.05)	RRMS	F:10(6.6)<br>NF:6(5.7)	EDSS≤2	F:17486(16736.5)<br>NF:7119.8(6571.5)	NR	NR	NR	NR
[94]	F:4(13)<br>NF:3(10)	F:38.5(7.6)<br>NF:37.8(9.8)	RRMS	F:9.3(7.3)<br>NF:7(6.2)	F:2.5(1-3)*<br>NF:1.5(0-3)*	NR	BDI<br>F:12.2(8.9)<br>NF:5.6(6.4)	NR	NR	NR
[51]	F:3(8)<br>NF:3(6)	F:38.5(3.2)<br>NF:35.9(7.8)	RRMS	MS:4.7(3.8)	F:0.4(0.5)<br>NF:0.3(0.5)	F:11(8,3)cm3<br>NF:17.2(8.1)cm3	BDI<br>F:8.3(3.2)<br>NF:6.2(4.3)	NR	9-HPT<br>F:19.2(3.2)<br>NF:19.1(2.1)	**F**:5 INF betaIb, 3 INF beta Ia, 1

										Natalizumab, 1 no untreated. **NF**:2 INF beta Ib, 6 INF beta Ia, 2 Cop-I, 1 untreated
[75]	F:8(30) NF:15(26)	F:34.5(20-58)* NF:30(17-54)*	RRMS	F:2(0-10)* NF:2(0-10)*	F:1.50(0-5.5)* NF:0.50(0-3.5)*	F:3.54(0.38-41.18)*mL NF:1.85(0.14-30.58)*mL	HADS≤10	**SDMT**: F:-0.65(-3.67-2.04) NF:-0.19(-2.46-2.22) **TAP-alertness**: F:0(-2-1.30) NF:0.10(-1.60-2) **PASAT-3**: F:-0.39(-4.74-1.1) NF:0.04(−3.23-1.31) **Bloc span**: F: −0.20 (−1.50 to 2.00) NF:0.20 (−1.60-2.40) **VLMT Dg 5**: F:0.13(−2.48-1.17) NF:0.36 (−1.44-1.17) **VLMT Dg7**: F: 0.23 (−2.74-1.28) NF:0.63(−1.40-1.43) **VLMT recognition**:	NR	F: 10 untreated, 10 interferons, 8 glatiramer acetate, 6 natalizumab, 3 fingolimod, 1 dimethylfumarate NF: 14 untreated, 15 interferons, 5 glatiramer acetate, 6 natalizumab, 1 fingolimod, 0 dimethylfumarate

								F:0.08(−4.01-0.94)<br>NF:0.39(−2.13 - 0.78)<br>**TMT-A**: F: −0.20 (−3.00-1.50)<br>**TMT-B**: F: −0.13 (−3.00-1.26)<br>**RWT lexical**: F: −0.30 (−2.00-1.60)<br>NF: 0.30 (−2.00-1.60)		
[95]	F:10<br>NF:9(17)	F:42(8)<br>NF:38(5)	RRMS	NR	F: 1.8(1-2.5)*<br>NF:1.5(1-1.5)*	NR	BDI<br>Data NR	NR	NR	NR
[83]	F:4(19)<br>NF:1(8)	F:40(32-42.5)*<br>NF: 36(32-39)*	RRMS	F:10(3.5-12.5)*<br>NF:6(2-12m)*	F:3(2.5-4)*<br>NF:2(2-2.5)*	F:3.53(1.66-11.72)*cm3<br>NF:3.94(1.98-4.83)*cm3	NR	NR	NR	NR

*Note. The data reported the mean (SD) value. *indicate the range instead of SD.*

Legend. BRB-N battery: Brief Repeatable Battery of Neuropsychological Tests; CA: central activation; CF: patients with cognitive fatigue; CNF: patients without cognitive fatigue; EC-AS: exteroceptive condition-accuracy score; EDSS: expanded disability status scale; EQ5D: EuroQol-5D quality of life questionnaire; ESS: Epworth sleepiness scale; F patients with fatigue; FSS: Fatigue Severity Scale; HADS: Hospital anxiety and depression scale; 9-HPT: 9-Hole peg Test; IC-AS: Interoceptive Condition-accuracy score; IFN: interferon; JTHFT: Jebsen-Taylor hand function test; MS: patients affected by multiple sclerosis; MSFC: Multiple Sclerosis functional composite; MSQOL-54: Multiple Sclerosis Quality of Life-54; MFIS: modified fatigue impact scale; MS: patients affected by Multiple Sclerosis; MSSS: Multiple sclerosis severity score; NF patients without fatigue; NR not reported; PSQI: Pittsburgh sleep quality index; TAS: Toronto alexithymia scale; tSTAI: Trait part of the Spielberger State Trait Anxiety Inventory; TWT: timed walk test; T25FW: timed 25-foot walk test; T1: time 1; T2: time 2.

1. Andreasen AK, Jakobsen J, Soerensen L, Andersen H, Petersen T, Bjarkam CR, Ahdidan J: **Regional brain atrophy in primary fatigued patients with multiple sclerosis**. *Neuroimage* 2010, **50**(2):608-615.
2. Bisecco A, Caiazzo G, d'Ambrosio A, Sacco R, Bonavita S, Docimo R, Cirillo M, Pagani E, Filippi M, Esposito F *et al*: **Fatigue in multiple sclerosis: The contribution of occult white matter damage**. *Mult Scler* 2016, **22**(13):1676-1684.
3. Bisecco A, Nardo FD, Docimo R, Caiazzo G, d'Ambrosio A, Bonavita S, Capuano R, Sinisi L, Cirillo M, Esposito F *et al*: **Fatigue in multiple sclerosis: The contribution of resting-state functional connectivity reorganization**. *Mult Scler* 2018, **24**(13):1696-1705.
4. Bernitsas E, Yarraguntla K, Bao F, Sood R, Santiago-Martinez C, Govindan R, Khan O, Seraji-Bozorgzad N: **Structural and Neuronal Integrity Measures of Fatigue Severity in Multiple Sclerosis**. *Brain Sci* 2017, **7**(8).
5. Calabrese M, Rinaldi F, Grossi P, Mattisi I, Bernardi V, Favaretto A, Perini P, Gallo P: **Basal ganglia and frontal/parietal cortical atrophy is associated with fatigue in relapsing-remitting multiple sclerosis**. *Mult Scler* 2010, **16**(10):1220-1228.
6. Campo CG, Salamone PC, Rodriguez-Arriagada N, Richter F, Herrera E, Bruno D, Cassara FP, Sinay V, Garcia AM, Ibanez A *et al*: **Fatigue in multiple sclerosis is associated with multimodal interoceptive abnormalities**. *Mult Scler J* 2019.
7. Chalah MA, Kauv P, Creange A, Hodel J, Lefaucheur JP, Ayache SS: **Neurophysiological, radiological and neuropsychological evaluation of fatigue in multiple sclerosis**. *Mult Scler Relat Disord* 2019, **28**:145-152.
8. Codella M, Rocca MA, Colombo B, Martinelli-Boneschi F, Comi G, Filippi M: **Cerebral grey matter pathology and fatigue in patients with multiple sclerosis: a preliminary study**. *J Neurol Sci* 2002, **194**(1):71-74.
9. Codella M, Rocca MA, Colombo B, Rossi P, Comi G, Filippi M: **A preliminary study of magnetization transfer and diffusion tensor MRI of multiple sclerosis patients with fatigue**. *J Neurol* 2002, **249**(5):535-537.
10. Cogliati Dezza I, Zito G, Tomasevic L, Filippi MM, Ghazaryan A, Porcaro C, Squitti R, Ventriglia M, Lupoi D, Tecchio F: **Functional and structural balances of homologous sensorimotor regions in multiple sclerosis fatigue**. *J Neurol* 2015, **262**(3):614-622.
11. Colombo B, Martinelli Boneschi F, Rossi P, Rovaris M, Maderna L, Filippi M, Comi G: **MRI and motor evoked potential findings in nondisabled multiple sclerosis patients with and without symptoms of fatigue**. *J Neurol* 2000, **247**(7):506-509.
12. Damasceno A, Damasceno BP, Cendes F: **Atrophy of reward-related striatal structures in fatigued MS patients is independent of physical disability**. *Mult Scler* 2016, **22**(6):822-829.
13. Derache N, Grassiot B, Mezenge F, Emmanuelle Dugue A, Desgranges B, Constans JM, Defer GL: **Fatigue is associated with metabolic and density alterations of cortical and deep gray matter in Relapsing-Remitting-Multiple Sclerosis patients at the earlier stage of the disease: A PET/MR study**. *Mult Scler Relat Disord* 2013, **2**(4):362-369.
14. Filippi M, Rocca MA, Colombo B, Falini A, Codella M, Scotti G, Comi G: **Functional magnetic resonance imaging correlates of fatigue in multiple sclerosis**. *Neuroimage* 2002, **15**(3):559-567.
15. Gobbi C, Rocca MA, Pagani E, Riccitelli GC, Pravata E, Radaelli M, Martinelli-Boneschi F, Falini A, Copetti M, Comi G *et al*: **Forceps minor damage and co-occurrence of depression and fatigue in multiple sclerosis**. *Mult Scler* 2014, **20**(12):1633-1640.

16. Gobbi C, Rocca MA, Riccitelli G, Pagani E, Messina R, Preziosa P, Colombo B, Rodegher M, Falini A, Comi G *et al*: **Influence of the topography of brain damage on depression and fatigue in patients with multiple sclerosis**. *Mult Scler* 2014, **20**(2):192-201.
17. Cruz Gomez AJ, Ventura Campos N, Belenguer A, Avila C, Forn C: **Regional brain atrophy and functional connectivity changes related to fatigue in multiple sclerosis**. *PLoS One* 2013, **8**(10):e77914.
18. Hanken K, Eling P, Kastrup A, Klein J, Hildebrandt H: **Integrity of hypothalamic fibers and cognitive fatigue in multiple sclerosis**. *Mult Scler Relat Disord* 2015, **4**(1):39-46.
19. Hanken K, Eling P, Klein J, Klaene E, Hildebrandt H: **Different cortical underpinnings for fatigue and depression in MS?** *Mult Scler Relat Disord* 2016, **6**:81-86.
20. Hanken K, Manousi A, Klein J, Kastrup A, Eling P, Hildebrandt H: **On the relation between self-reported cognitive fatigue and the posterior hypothalamic-brainstem network**. *Eur J Neurol* 2016, **23**(1):101-109.
21. Hanken K, Francis Y, Kastrup A, Eling P, Klein J, Hildebrandt H: **On the role of the amygdala for experiencing fatigue in patients with multiple sclerosis**. *Mult Scler Relat Disord* 2018, **20**:67-72.
22. Hidalgo de la Cruz M, d'Ambrosio A, Valsasina P, Pagani E, Colombo B, Rodegher M, Falini A, Comi G, Filippi M, Rocca MA: **Abnormal functional connectivity of thalamic sub-regions contributes to fatigue in multiple sclerosis**. *Mult Scler* 2018, **24**(9):1183-1195.
23. Marchesi O, Vizzino C, Meani A, Conti L, Riccitelli GC, Preziosa P, Filippi M, Rocca MA: **Fatigue in multiple sclerosis patients with different clinical phenotypes: a clinical and magnetic resonance imaging study**. *Eur J Neurol* 2020, **27**(12):2549-2560.
24. Morgante F, Dattola V, Crupi D, Russo M, Rizzo V, Ghilardi MF, Terranova C, Girlanda P, Quartarone A: **Is central fatigue in multiple sclerosis a disorder of movement preparation?** *Journal of Neurology* 2011, **258**(2):263-272.
25. Niepel G, Tench Ch R, Morgan PS, Evangelou N, Auer DP, Constantinescu CS: **Deep gray matter and fatigue in MS: a T1 relaxation time study**. *J Neurol* 2006, **253**(7):896-902.
26. Palotai M, Mike A, Cavallari M, Strammer E, Orsi G, Healy BC, Schregel K, Illes Z, Guttmann CR: **Changes to the septo-fornical area might play a role in the pathogenesis of anxiety in multiple sclerosis**. *Mult Scler* 2018, **24**(8):1105-1114.
27. Palotai M, Cavallari M, Koubiyr I, Morales Pinzon A, Nazeri A, Healy BC, Glanz B, Weiner HL, Chitnis T, Guttmann CR: **Microstructural fronto-striatal and temporo-insular alterations are associated with fatigue in patients with multiple sclerosis independent of white matter lesion load and depression**. *Mult Scler* 2019:1352458519869185.
28. Palotai M, Nazeri A, Cavallari M, Healy BC, Glanz B, Gold SM, Weiner HL, Chitnis T, Guttmann CRG: **History of fatigue in multiple sclerosis is associated with grey matter atrophy**. *Sci Rep* 2019, **9**(1):14781.
29. Palotai M, Small C, Makris N, Somes NG, Pinzon AM, Rathi Y, Marzullo A, Levitt JJ, Bakshi R, Chitnis T *et al*: **Microstructural Changes in the Left Mesocorticolimbic Pathway are Associated with the Comorbid Development of Fatigue and Depression in Multiple Sclerosis**. *J Neuroimaging* 2021.
30. Pellicano C, Gallo A, Li X, Ikonomidou VN, Evangelou IE, Ohayon JM, Stern SK, Ehrmantraut M, Cantor F, McFarland HF *et al*: **Relationship of cortical atrophy to fatigue in patients with multiple sclerosis**. *Arch Neurol* 2010, **67**(4):447-453.

31. Pravata E, Zecca C, Sestieri C, Caulo M, Riccitelli GC, Rocca MA, Filippi M, Cianfoni A, Gobbi C: **Hyperconnectivity of the dorsolateral prefrontal cortex following mental effort in multiple sclerosis patients with cognitive fatigue**. *Mult Scler* 2016, **22**(13):1665-1675.
32. Riccitelli G, Rocca MA, Forn C, Colombo B, Comi G, Filippi M: **Voxelwise assessment of the regional distribution of damage in the brains of patients with multiple sclerosis and fatigue**. *AJNR Am J Neuroradiol* 2011, **32**(5):874-879.
33. Rocca MA, Agosta F, Colombo B, Mezzapesa DM, Falini A, Comi G, Filippi M: **fMRI changes in relapsing-remitting multiple sclerosis patients complaining of fatigue after IFNbeta-1a injection**. *Hum Brain Mapp* 2007, **28**(5):373-382.
34. Rocca MA, Gatti R, Agosta F, Broglia P, Rossi P, Riboldi E, Corti M, Comi G, Filippi M: **Influence of task complexity during coordinated hand and foot movements in MS patients with and without fatigue. A kinematic and functional MRI study**. *J Neurol* 2009, **256**(3):470-482.
35. Rocca MA, Absinta M, Valsasina P, Copetti M, Caputo D, Comi G, Filippi M: **Abnormal cervical cord function contributes to fatigue in multiple sclerosis**. *Mult Scler* 2012, **18**(11):1552-1559.
36. Rocca MA, Parisi L, Pagani E, Copetti M, Rodegher M, Colombo B, Comi G, Falini A, Filippi M: **Regional but not global brain damage contributes to fatigue in multiple sclerosis**. *Radiology* 2014, **273**(2):511-520.
37. Rocca MA, Meani A, Riccitelli GC, Colombo B, Rodegher M, Falini A, Comi G, Filippi M: **Abnormal adaptation over time of motor network recruitment in multiple sclerosis patients with fatigue**. *Mult Scler* 2016, **22**(9):1144-1153.
38. Roelcke U, Kappos L, Lechner-Scott J, Brunnschweiler H, Huber S, Ammann W, Plohmann A, Dellas S, Maguire RP, Missimer J *et al*: **Reduced glucose metabolism in the frontal cortex and basal ganglia of multiple sclerosis patients with fatigue: a 18F-fluorodeoxyglucose positron emission tomography study**. *Neurology* 1997, **48**(6):1566-1571.
39. Sander C, Eling P, Hanken K, Klein J, Kastrup A, Hildebrandt H: **The Impact of MS-Related Cognitive Fatigue on Future Brain Parenchymal Loss and Relapse: A 17-Month Follow-up Study**. *Front Neurol* 2016, **7**:155.
40. Sepulcre J, Masdeu JC, Goni J, Arrondo G, Velez de Mendizabal N, Bejarano B, Villoslada P: **Fatigue in multiple sclerosis is associated with the disruption of frontal and parietal pathways**. *Mult Scler* 2009, **15**(3):337-344.
41. Specogna I, Casagrande F, Lorusso A, Catalan M, Gorian A, Zugna L, Longo R, Zorzon M, Naccarato M, Pizzolato G *et al*: **Functional MRI during the execution of a motor task in patients with multiple sclerosis and fatigue**. *Radiol Med* 2012, **117**(8):1398-1407.
42. Stefancin P, Govindarajan ST, Krupp L, Charvet L, Duong TQ: **Resting-state functional connectivity networks associated with fatigue in multiple sclerosis with early age onset**. *Mult Scler Relat Disord* 2019, **31**:101-105.
43. Svolgaard O, Andersen KW, Bauer C, Madsen KH, Blinkenberg M, Selleberg F, Siebner HR: **Cerebellar and premotor activity during a non-fatiguing grip task reflects motor fatigue in relapsing-remitting multiple sclerosis**. *PLoS One* 2018, **13**(10):e0201162.
44. Tartaglia MC, Narayanan S, Francis SJ, Santos AC, De Stefano N, Lapierre Y, Arnold DL: **The relationship between diffuse axonal damage and fatigue in multiple sclerosis**. *Arch Neurol* 2004, **61**(2):201-207.

45. Tedeschi G, Dinacci D, Lavorgna L, Prinster A, Savettieri G, Quattrone A, Livrea P, Messina C, Reggio A, Servillo G *et al*: **Correlation between fatigue and brain atrophy and lesion load in multiple sclerosis patients independent of disability**. *J Neurol Sci* 2007, **263**(1-2):15-19.
46. Tellez N, Alonso J, Rio J, Tintore M, Nos C, Montalban X, Rovira A: **The basal ganglia: a substrate for fatigue in multiple sclerosis**. *Neuroradiology* 2008, **50**(1):17-23.
47. Tomasevic L, Zito G, Pasqualetti P, Filippi M, Landi D, Ghazaryan A, Lupoi D, Porcaro C, Bagnato F, Rossini P *et al*: **Cortico-muscular coherence as an index of fatigue in multiple sclerosis**. *Mult Scler* 2013, **19**(3):334-343.
48. Wilting J, Rolfsnes HO, Zimmermann H, Behrens M, Fleischer V, Zipp F, Groger A: **Structural correlates for fatigue in early relapsing remitting multiple sclerosis**. *Eur Radiol* 2016, **26**(2):515-523.
49. Zaini WH, Giuliani F, Beaulieu C, Kalra S, Hanstock C: **Fatigue in Multiple Sclerosis: Assessing Pontine Involvement Using Proton MR Spectroscopic Imaging**. *PLoS One* 2016, **11**(2):e0149622.
50. Zellini F, Niepel G, Tench CR, Constantinescu CS: **Hypothalamic involvement assessed by T1 relaxation time in patients with relapsing-remitting multiple sclerosis**. *Mult Scler* 2009, **15**(12):1442-1449.
